# Analysis of clinical characteristics and risk factors of community-acquired pneumonia complicated by parapneumonic pleural effusion in elderly patients

**DOI:** 10.1186/s12890-023-02649-4

**Published:** 2023-09-20

**Authors:** Mingmei Zhong, Ruiqin Ni, Huizhen Zhang, Yangyang Sun

**Affiliations:** 1 The Third Affiliated Hospital of Anhui Medical University, Hefei, 230061 China; 2 Bengbu Medical College, Bengbu, 233030 China

**Keywords:** Community acquired pneumonia, Parapneumonic pleural effusion, Elderly, Clinical characteristics, Risk factors

## Abstract

**Background:**

Community-acquired pneumonia (CAP) patients usually present with parapneumonic pleural effusion (PPE), which complicates the treatment of pneumonia. This study aims to investigate the clinical characteristics and risk factors of elderly CAP patients hospitalised with PPE.

**Methods:**

The clinical data of 132 elderly patients with CAP were retrospectively analysed. A total of 54 patients with PPE (PPE group) and 78 patients without PPE (NPPE group) were included in this study. Clinical data, laboratory examinations, treatments and other relevant indicators were collected. Univariate analysis and multivariate logistic regression analysis will be used to explore the possible risk factors for PPE.

**Results:**

The proportion of PPE in elderly patients with CAP was 40.9%. PPE patients were significantly more likely to be older, have comorbid neurological diseases, experience chest tightness, and have a lasting fever (*P* < 0.05). In contrast to NPPE patients, the total number of lymphocytes, serum albumin and blood sodium levels in the PPE group were significantly lower (*P* < 0.05). The blood D-dimer, C-reactive protein and CURB-65 score of PPE patients were significantly higher (*P* < 0.05) than those of NPPE patients. Multivariate logistic regression identified chest tightness (OR = 3.964, 95% CI: 1.254–12.537, *P* = 0.019), long duration of fever (OR = 1.108, 95%CI: 1.009–1.217, *P* = 0.03), low serum albumin (OR = 0.876, 95%CI: 0.790– 0.971, *P* = 0.012) or low blood sodium (OR = 0.896, 95%CI: 0.828–0.969, *P* = 0.006) as independently associated with the development of parapneumonic pleural effusion in the elderly.

**Conclusion:**

This study has identified several clinical factors, such as chest tightness, long duration of fever, low serum albumin, and low blood sodium, as risk factors for the development of pleural effusion in elderly patients with CAP. Early identification and prompt management of these patients can prevent inappropriate treatment and reduce morbidity and mortality.

**Supplementary Information:**

The online version contains supplementary material available at 10.1186/s12890-023-02649-4.

## Introduction

Parapneumonic pleural effusion (PPE) refers to exudative pleural effusion secondary to pneumonia, lung abscess and bronchiectasis. It is a frequent complication of pneumonia [1]. PPE, which is secondary to endothelial injury induced by activated neutrophils occurs due to increased capillary permeability [2, 3]. Previous studies have shown that the development of pleural effusion in correlation with many factors, not only related to pulmonary and pleural infections, pathogenic bacteria and various inflammations, but also related to the patient’s health status and underlying diseases [4, 5]. A national multicenter, retrospective, observational cross-sectional study initiated by the CAP-China network (clinical trial registry number: NCT02489578) select 13 hospitals in different regions of China from January 1, 2014 to December 31, 2014. Of the 4 781 patients with CAP, 1 169 (24.5%) were PE patients, with a median age of 70 years, and more males than females, having smoking, alcoholism, inhalation factors, long-term bed rest, complicated with underlying diseases and complications, such as cardiovascular disease, cerebrovascular disease and so on [4]. In India, the etiology of 2906 patients with parapneumonic pleural effusion were analyzed, of which 459 (15.8%) samples were culture positive [6]. The most frequent Gram-negative organisms were Acinetobacter spp. (27.7%), Pseudomonas aeruginosa (23.9%) and Klebsiella spp. (12.6%). Staphylococcus aureus (9.6%) was the most frequent Gram-positive organism. Most of the pathogens showed resistance to multiple antibiotic agents. In addition, in the Spanish and UK, a history of alcohol abuse or intravenous drug use have been reported to be associated with development of complicated parapneumonic effusion or empyema [7, 8].

The literature reports that 15%–44% of hospitalised patients with community-acquired pneumonia (CAP) have PPE [9], and approximately 5% of pneumonia patients have complicated parapneumonic pleural effusion and pleural parapneumonic empyema. Approximately 33% of patients with PPE who fail to respond to antibiotics and chest drainage require surgical treatment [10]. Concomitant PPE complicates the treatment of pneumonia [11], and the course and aggressiveness of parapneumonic pleural effusions vary widely. Therefore, understanding their progression is important. In addition to increased mortality, complicated parapneumonic pleural effusions and empyema often require long-term treatment, longer hospital stays and interventions. Therefore, identifying and managing these patients in a timely manner are important [12, 13].

In recent years, with the development of an aging society, the incidence of CAP has increased in the elderly, and PPE has become more common [14]. However, few data on PPE in elderly patients with CAP are available. This study retrospectively analysed the clinical data of 132 elderly patients with CAP to identify the risk factors for PPE. To better prevent the occurrence of pleural effusion alongside pneumonia, treatment should be initiated earlier to avoid further development of the disease and improve the survival rate.

## Data and methods

### Study design and inclusion and exclusion criteria

**Fig. 1:**
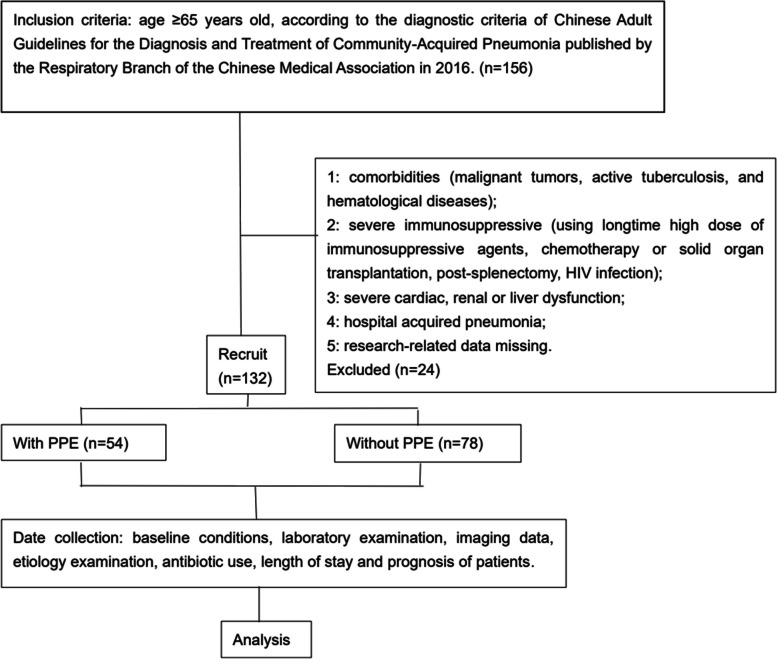
Group assignment

A retrospective case–control study was conducted at the Department of Respiratory and Critical Care Medicine of the Third Affiliated Hospital of Anhui Medical University. Clinical data from 132 elderly patients diagnosed with CAP were collected between January 2019 and December 2019. Subjects were included in the study if they fulfilled the following criteria: Age ≥ 65 years old, according to the diagnostic criteria of *Chinese Adult Guidelines for the Diagnosis and Treatment of Community-Acquired Pneumonia* published by the Respiratory Branch of the Chinese Medical Association in 2016 [15]. Subjects with any of the following were excluded based on the criteria [16]: comorbidities (malignant tumours, active tuberculosis and haematological diseases); severe immunosuppression (using long-term high doses of immunosuppressive agents, chemotherapy or solid organ transplantation, post-splenectomy, HIV infection); severe cardiac, renal or liver dysfunction; hospital-acquired pneumonia; and research-related data missing. Depending on the presence of pleural effusion or not, patients were divided into the PPE group (case group) and NPPE group (control group) (Fig. 1). The Clinical Research Ethics Committee of the Third Affiliated Hospital of Anhui Medical University approved this study [LUN Research Grant No. 2020 (20)].

### Methods

Data collection: We retrospectively extracted the following patient data by reviewing medical records, general conditions, underlying diseases, symptoms, signs, laboratory examinations, imaging data, aetiology examinations, antibiotic use, length of stay and patient prognosis.

Definitions: The disease course prior to hospital admission refers to the time from the discovery of clinical symptoms to admission. ‘Quit smoking’ refers to having quit smoking for at least 6 months. ‘Consciousness change’ indicates new onset confusion. ‘Chest tightness’ includes symptoms, such as breathlessness, rapidly progressive dyspnoea (shortness of breath), use of accessory respiratory muscles, laboured breathing and extreme wheezing. Patient prognosis is defined as 28-d all-cause mortality.

Record the CURB-65 score. The CURB-65 score consists of 5 points: confusion, blood urea nitrogen > 7 mmol/L, respiratory rate > 30 breaths/min, systolic blood pressure < 90 mmHg and/or diastolic blood pressure ≤ 60 mmHg, and age ≥ 65 years [17, 18]. One point is assigned for each item above.

### Statistical methods

All data were analysed using the Statistical Package for Social Sciences (SPSS) version 25. Normally distributed quantitative data are presented as means ± standard deviations (SDs) and compared using the unpaired t-test. Non-normally distributed quantitative data were expressed as medians (interquartile ranges) [M (Q1, Q3)], and non-parametric tests (Mann–Whitney U test) were used. Categorical data are presented as numbers and percentages and compared using Pearson's Chi-square test. All variables that were statistically significant in the univariate analysis with a *p*-value < 0.05 were entered into a multivariable model using a stepwise approach. A logistic regression model was performed in order to evaluate risk factors of parapneumonic pleural effusion in elderly patients. A *p*-value of < 0.05 was considered statistically significant for each analysis.

## Results

### Patient characteristics

Depending on imaging data (e.g. chest* X*-ray, chest CT or chest B-ultrasound), patients were divided into the PPE group, consisting of 54 cases, including 34 males and 20 females. The age of the patients ranged from 69 to 96 years (average 81.7 ± 7.1 years). Amongst the 54 PPE patients, 42 were cases of unilateral pleural effusion and 12 were cases of bilateral pleural effusion. Out of these numbers, 41 patients underwent thoracentesis and drainage, and all of the drainage samples were exudated, with a total volume ranging from approximately 400 to 1500 ml. A total of 13 patients did not undergo puncture due to either a low pleural effusion volume or high puncture risk. The NPPE group consisted of 78 cases, including 41 males and 37 females. The age of the patients in this group ranged from 65 to 99 years (average 78.4 ± 8.5 years). CAP patients hospitalised with PPE were more likely to be older and had comorbid neurological diseases, such as stroke, Parkinson's disease and senile dementia). The sex ratio (*χ*^*2*^ = 1.41, *P* = 0.24), smoking status (*χ*^*2*^ = 0.002, *P* = 0.96; *χ*^*2*^ = 0.09, *P* = 0.76; *χ*^*2*^ = 0.82, *P* = 0.78, respectively), previous hypertension (*χ*^*2*^ = 0.92, *P* = 0.34), coronary heart disease (*χ*^*2*^ = 0.38, *P* = 0.54), chronic cardiac insufficiency (*χ*^*2*^ = 0.54, *P* = 0.46), chronic obstructive pulmonary disease (*χ*^*2*^ = 1.77, *P* = 0.0.18), diabetes (*χ*^*2*^ = 0.04, *P* = 0.84) and renal insufficiency (*χ*^*2*^ = 2.85, *P* = 0.09) in the two groups were similar (Table 1).

**Table 1:** Characteristics of patients between the two groups [n (%)/($\overline{x }$±s)]

Project	NPPE group (*n* = 78)	PPE group (*n* = 54)	*t* / *χ*^2^value	*P*-value
Gender (Male/female)	41/37	34/20	1.406	0.236
Age (yr)	78.4 ± 8.5	81.7 ± 7.1	2.351	0.020
Smoking status				
Never smoking	48 (61.54)	33 (61.11)	0.002	0.960
Quit smoking	17 (21.79)	13 (24.07)	0.094	0.759
Still smoking	13 (16.67)	8 (14.81)	0.082	0.775
Basic diseases				
Hypertension	47 (60.26)	28 (51.85)	0.919	0.338
Coronary heart disease	10 (12.82)	9 (16.67)	0.383	0.536
Chronic cardiac insufficiency	7 (8.97)	7 (12.96)	0.535	0.464
Chronic obstructive pulmonary disease	7 (8.97)	9 (16.67)	1.773	0.183
Diabetes	20 (25.64)	13 (24.07)	0.042	0.838
Insufficiency of kidney function	2 (2.56)	5 (9.26)	2.848	0.091
Diseases of the nervous system	20 (25.64)	23 (42.59)	4.175	0.041
Number of underlying diseases (species)	1.6 ± 1.1	1.9 ± 1.1	1.520	0.131

*t*-test: Age, Number of underlying diseases; *χ*^*2*^ test: Gender; Smoking status; Basic diseases

### Comparison of clinical features between the two groups

The incidence of chest tightness and duration of fever in the PPE group were significantly higher than those in the NPPE group (*P* < 0.05). The CURB-65 score in the PPE group was significantly higher than that in the NPPE group (*t* = 3.543, *P* = 0.001). No significant differences were observed in the proportion of fever (*χ*^*2*^ = 0.01, *P* = 0.93), cough (*χ*^*2*^ = 0.01, *P* = 0.94), chest pain (χ^*2*^ = 0.92, *P* = 0.34), altered consciousness (new onset confusion; *χ*^*2*^ = 1.63, *P* = 0.20), maximum body temperature (*χ*^*2*^ = 0.15, *P* = 0.89) during the course of the disease and the number of days of illness before admission (*χ*^*2*^ = 02.75, *P* = 0.0.10; *χ*^*2*^ = 0.47, *P* = 0.49; *χ*^*2*^ = 1.01, *P* = 0.31, respectively) between the two groups. The results are shown in Table 2.

**Table 2:** Comparison of relevant clinical features between the two groups [n (%)/($\overline{x }$±s)]

project	NPPE group (*n* = 78)	PPE Group (*n* = 54)	*t* / χ^2^value	*P*-value
Fever	54 (69.23)	37 (68.52)	0.008	0.931
Maximum body temperature (℃)	38.64 ± 0.69	38.66 ± 0.62	0.151	0.881
Duration of fever (d)	6.33 ± 3.99	8.89 ± 6.88	2.242	0.027
Cough	69 (88.46)	48 (88.89)	0.006	0.939
Chest pain	5 (6.41)	6 (11.11)	0.923	0.337
chest tightness	16 (20.51)	28 (51.85)	14.103	0.000
Consciousness change	4 (5.13)	6 (11.11)	1.631	0.202
Course of illness before admission				
1 ~ 4 d	25 (32.05)	25 (46.30)	2.752	0.097
5 ~ 10 d	36 (46.15)	21 (38.89)	0.468	0.494
> 10d	17 (21.79)	8 (14.81)	1.013	0.314
CURB-65 score	1.6 ± 0.9	2.2 ± 1.0	3.543	0.001

Fever: T > 37.4 ℃; *t*-test: Maximum body temperature, Duration of fever, CURB-65 score; *χ*^*2*^ test: Cough, Chest pain, Chest distress, Consciousness change, Course of illness before admission

### Laboratory examination of patients in two groups

The total number of lymphocytes, serum albumin and blood sodium levels in the PPE group were significantly lower than those in the NPPE group (P < 0.05). Blood D-dimer (*t* = 2.25, *P* = 0.02) and C-reactive protein (CRP) (*t* = 2.38, *P* = 0.02) in the PPE group were significantly higher than those in the NPPE group. No significant difference was observed in other indexes between the two groups (*P* > 0.05). The results are shown in Table 3.

**Table 3:** Comparison of laboratory test results between the two groups [median (IQR)/(($\overline{x }$±s)]

Project	NPPE group (*n* = 78)	PPE Group (*n* = 54)	*t/Z* value	*P*-value
Total white blood cell count (× 10^9^/ L)	8.78 (5.85, 11.73)	9.46 (6.71, 11.74)	1.176	0.240
The total number of neutrophils (× 10^9^/ L)	6.77 (3.77, 9.22)	7.33 (5.13, 10.28)	1.673	0.094
Total number of lymphocytes (× 10^9^/ L)	1.10 (0.83, 1.59)	0.95 (0.64, 1.16)	2.634	0.008
Hemoglobin (g/L)	121.86 ± 17.48	116.41 ± 21.76	1.592	0.114
Platelets (× 10^9^/ L)	209.72 ± 89.64	209.48 ± 81.02	0.015	0.988
Serum albumin (g/L)	37.03 ± 5.21	33.16 ± 7.15	3.597	< 0.001
Blood urea nitrogen (mmol/L)	7.34 ± 5.49	8.35 ± 4.95	0.181	0.282
Serum creatinine (µmol/L)	86.35 ± 52.93	83.73 ± 43.92	0.299	0.765
Blood sodium (mmol/L)	140.21 ± 5.66	137.69 ± 7.79	2.153	0.033
D-dimer (mg/L)	0.96 (0.51, 1.97)	1.43 (0.85, 3.24)	2.254	0.024
Fibrinogen (g/L)	4.97 ± 1.59	4.92 ± 1.63	0.174	0.862
CRP (mg/L)	68.84 ± 53.94	95.32 ± 73.89	2.380	0.019
Procalcitonin (ng/mL)	2.44 ± 7.30	1.36 ± 2.83	1.046	0.297

*t*-test: Haemoglobin, Serum albumin, Blood urea nitrogen, Serum creatinine, Fibrinogen, CRP, Procalcitonin. *Z*-test: D-dimer, Total white blood cell count, The total number of neutrophils, Total number of lymphocytes

### Pathogen distribution

In these recruited patients, 113 underwent etiological examination, including sputum smear and culture, alveolar perfusion fluid, pleural effusion, blood culture and serum detection of pathogen antibodies. The aetiological submission rates in the PPE and NPPE groups were 87.1% (47/54) and 84.6% (66/78), respectively. In the NPPE group, 22 results were positive, including 7 *Gram-negative bacilli* (the bacteria species could not be identified) in sputum smear, 7 *Candida albicans*, 2 *Pseudomonas aeruginosa*, 1 *Escherichia coli*, and 1 *Klebsiella ornithinolyticus* in sputum culture, 1 *Escherichia coli* in blood culture, and 3 Mycoplasma pneumoniae-specific antibody IgM. In the PPE group, 23 positive results were present (42.6%, including 2 mixed infections with two pathogens), including 5 *Gram-negative bacilli* (the bacteria species could not be identified) in sputum smear, 4 *Candida albicans*, 2 *Candida parapsilosis*, 2 *Staphylococcus aureus*, 2 *Serratia marcescens*, 2 *Acinetobacter baumannii*, 1 *Pseudomonas aeruginosa*, 1 *Klebsiella pneumoniae*, 1 *Escherichia coli* and 1 *Haemophilus influenzae* in sputum culture; 1 *Escherichia coli*, 1 *Staphylococcus aureus*, and 1 *Staphylococcus haemolytic* in blood culture; and 1 *Streptococcus agalactiae* in pleural effusion culture.

### Antibiotic use and clinical prognosis

All patients with CAP received intravenous antibiotic therapy, including β-lactam, β-lactamase inhibitors, quinolones, macrolides, carbapenems, glycopeptides, and triazole. NPPE patients were mostly treated with a single drug, with second-generation cephalosporins, third-generation cephalosporins or β-lactamase inhibitors being the main choices. The utilisation rate of carbapenems or glycopeptides was 10.3% (8/78), and the proportion of combined drug use was 19.2% (15/78). PPE patients were mostly treated with β-lactamase inhibitors, with a utilisation rate of carbapenems or glycopeptides at 25.9% (14/54), and the proportion of combined drug use was 22.2% (12/54). When compared with NPPE patients, the use rate of carbapenems or glycopeptides was higher (*χ*^*2*^ = 5.641, *P* = 0.018), the length of hospital stay was longer (*t* = 2.073, *P* = 0.04), and the in-hospital mortality of PPE patients was significantly higher (*χ*^*2*^ = 12.551, *P* < 0.001). The results are shown in Table 4.

**Table 4:** Comparison of antibiotic use and clinical prognosis between the two groups [n (%)/($\overline{x }$±s)]

project	NPPE group (*n* = 78)	PPE Group (*n* = 54)	*t* /* χ*^*2*^value	*P*-value
Antibiotic Use				
Carbapenems or glycopeptides	8 (10.3)	14 (25.9)	5.641	0.018
Combination	15 (19.2)	12 (22.2)	0.175	0.675
Length of hospital stay (d)	11.4 ± 4.6	13.6 ± 7.6	2.073	0.04
In-hospital death	6 (7.69)	17 (31.48)	12.551	< 0.001

*t*-test: Length of hospital stay;* χ*^*2*^test: Antibiotic use, Combination, In-hospital death

### Multivariate regression analysis of CAP with pleural effusion in elderly patients

In the above univariate analysis, several significant factors were identified as independent variables, including age, neurological disease (yes/no), symptoms of chest tightness (yes/no), duration of fever, total lymphocyte count, serum albumin, serum sodium, D-dimer, CRP, and CURB-65 score, with the dependent variables assigned as follows: Yes = 1, No = 0. Multivariate Logistic regression analysis was then performed. The results display that chest tightness (OR = 3.96, 95% CI 1.25–12.54, *P* = 0.02), long duration of fever (OR = 1.11, 95% CI 1.01– 1.22, *P* = 0.03), low serum albumin (OR = 0.88, 95% CI 0.79–0.97, *P* = 0.01) and low blood sodium (OR = 0.90, 95% CI 0.83–0.97, *P* = 0.01) were significant risk factors for elderly CAP patients combined with PPE. The results are shown in Table 5.

**Table 5:** Multivariate Logistic regression analysis of PPE in elderly patients with CAP

Variable	B value	SE	Wald χ^2^value	*P*-value	OR value (95%CI)
Duration of fever	0.102	0.048	4.578	0.032	1.108 (1.009 ~ 1.217)
Chest tightness	1.377	0.587	5.497	0.019	3.964 (1.254 ~ 12.537)
Serum albumin	0.133	0.053	6.348	0.012	0.876 (0.790 ~ 0.971)
Serum sodium	0.110	0.040	7.533	0.006	0.896 (0.828 ~ 0.969)

## Discussion

CAP is becoming an increasing problem in the elderly, with literature reporting that approximately 45% to 50% of all CAP hospitalisations occur in patients 65 yrs of age or older [19]. Early antibiotic treatment is crucial to prevent the development of a PPE [2]. Poor clinical outcomes and increased mortality can be associated with the development of parapneumonic effusions [20]. Research shows that hypoalbuminaemia, hyponatraemia and an elevated CRP have been identified as independent risk factors for mortality [8, 21]. In this study, a retrospective analysis of the clinical data of 132 elderly patients with CAP in our hospital showed that the proportion of elderly patients with CAP combined with PPE was 40.9%, and the in-hospital mortality of pneumonia patients with PPE was significantly higher than those of patients with pneumonia alone. Therefore, the study of clinical characteristics of elderly patients with CAP combined with PPE is conducive to early recognition, diagnosis and treatment.

In addition to factors, such as inflammation and virulence characteristics due to direct bacterial invasion of the lungs and pleural cavity, patients' own conditions and underlying diseases contribute to the pathophysiology development of PPE. In this study, older adults with CAP had more underlying diseases, with 87.1% of the patients having at least one disease. The presence of underlying disease is an important risk factor for pneumonia in old age, leading to reduced resistance to infection and an increased risk of pneumonia and death [22]. Previous studies have highlighted diabetes mellitus, malignancy, chronic alcohol intake, chronic lung disease, immunosuppressive status and inhalation as the most common comorbidities in patients with PPE [23, 24]. However, in this study, the proportion of PPE combined with neurological diseases was higher, and it was associated with a significantly increased risk of inhalational pneumonia in older patients with neurological diseases, such as stroke, dementia and Parkinson's disease [25]. For the first time after aspiration pneumonia, the 1-month mortality was 23.9% in patients with Parkinson's disease, and about two-thirds of the patients died within 1 year after the first episode of aspiration [26, 27]. The PPE group showed high in-hospital mortality in this study. Considering these patients had significantly older ages, long-term bedridden status, cognitive impairment, or swallowing dysfunction, their onset was primarily associated with aspiration pneumonia or more lung infiltrates. The diagnosis and treatment of these patients were often delayed after onset, or their families had a poor willingness to undergo invasive operations, such as endotracheal intubation in treatment. Therefore, PPE patients had a higher in-hospital mortality rate than NPPE. Additionally, the PPE group did not have a high proportion of diabetes but considering the relatively small sample size, we acknowledge potential selection bias.

Regarding aetiology, in this study, 42.6% of patients in the PPE group were detected with pathogenic bacteria, with the order being *G*^*–*^*Bacillus*, *Candida* and *Staphylococcus*. The main infections of *G–Bacillus* were *Enterobacteriaceae*, such as *E. coli* and *Klebsiella pneumoniae*, whilst one case of pleural effusion was cultured as *S. agalactiae*. A meta-analysis by Hassan et al. [28] showed that the most common aerobic isolation bacteria in pleural effusion culture were *S. aureus* (20.7%), *Streptococcus aerophylus* group (18.7%), *Pseudomonas* (17.6%), *Enterobacteriaceae* (11.9%), *Streptococcus pneumoniae* (10.8%), *Klebsiella* (10.7%), *Acinetobacter* (5%), and *coagulase*-*negative Staphylococcus* (4.5%). The distribution of pathogenic bacteria in this study differed from that in the literature. On the one hand, the pathogenic specimens were different, and on the other hand, the positive rate of pathogenic bacteria was not high due to empirical antibiotic therapy before specimen submission for examination. With the advent of whole-genome second-generation sequencing, we may overcome some of the shortcomings of standard microscopy and culture techniques, and the impact of antibiotic use will also decrease. This way, more or less common pathogens will be identified, which will be more helpful for clinical decision-making.

The treatment of PPE requires prompt resolution of intrathoracic infection and antimicrobial therapy, which should be guided by sensitivity to specific pathogens [29]. In cases of culture-negative PPE, empirical antibiotic use should be based on local pathogenic distribution, drug resistance and antibiotic management policies. The British Thoracic Society (BTS) [30] and the American Association for Thoracic Surgery [31] advise the use of broad-spectrum antibiotics that cover Gram-*positive*, *Gram-negative* and *anaerobic* bacteria, such as β-lactamase inhibitors, third-generation cephalosporins and carbapenems. For hospitalised patients with CAP, the use of β-lactam alone or in combination with doxycycline, minocycline, macrolides, or respiratory quinolones is recommended. Elderly patients aged ≥ 65 years or with underlying diseases should be considered for the possibility of Enterobacteriaceae bacterial infection, and they can be treated with cephamycin, third-generation cephalosporin combined with β-lactamase inhibitors, or other empirical treatments [14]. Notably, the elderly have strong resistance to penicillin and should avoid its use, so beta-lactamase antibiotics are preferred [32]. Consistent with these recommendations, due to the high drug resistance rate of Macrolide in China, it is generally not considered for treatment. In this study, the use of antibiotics in the PPE group was mainly β-lactam inhibitors, and the utilisation rate of carbapenems or glycopeptides was 10.3% (14/54), which was significantly higher than that in the NPPE group. This difference can be attributed to the pathophysiological characteristics of the PPE group and the severity of patients' disease.

Clinically, pleurisy often leads to chest pain, but it is uncommon in the PPE group (11.1%), and 6.41% of patients still have chest pain without effusion. Therefore, the diagnosis of pleurisy cannot be solely based on chest pain. Dyspnea is the most common manifestation of pleural effusion, and the severity of dyspnea does not clearly correlate with the amount of effusion, possibly related to changes in gas exchange, respiratory mechanics, muscle function and hemodynamics, which are caused by pleural effusion [33, 34]. Although 20.51% of the NPPE patients experienced chest tightness, the proportion of chest tightness and dyspnea in the PPE group was higher (51.85%), and chest tightness was an independent risk factor for PPE. Therefore, for elderly CAP patients with chest tightness, being alert whether pleural effusion is combined is necessary. Our study demonstrated that PPE patients easily presented long-term fever, suggesting that body temperature, as a clinical marker of inflammation, lasted longer in patients with pleural effusion. the persistence of fever in patients with pneumonia can complicate the conditions, and suggests that inflammation persists [35].

Inflammatory markers are significantly elevated in PPE and empyema patients because of the persistence of pleural inflammation [36]. The results of this study showed no significant differences in peripheral white blood cells count, neutrophil count and PCT between the PPE and the NPPE patients, whilst the CRP level in the PPE group was significantly higher than that in the NPPE group. As a classic inflammatory marker, CRP is widely used in the diagnosis of infectious diseases [37]. In a previous study, pleural fluid CRP levels can be used to distinguish between parapneumonic effusions and other types of exudative effusions [38]. CRP levels < 0.64 mg/dL are likely to indicate a pleural effusion from congestive heart failure, whereas levels ≥ 1.38 mg/dL are suggestive of an infectious aetiology. The study of Petrusevska–Marinkovic [24] suggested that the CRP in the complex PPE patients was significantly higher than that in simple PPE patients [(231.79 ± 112.2) mg/L vs. (163.8 ± 147.9) mg/L, *P* < 0.01], and both were significantly higher than those in CAP patients without effusion [(139.48 ± 105.7) mg/L, *P* < 0.01]. Patients whose CRP does not decline with treatment during the course of the disease are at significantly higher risk for complex PPE or empyema. D-dimer is an objective biomarker for reflecting coagulation and fibrinolysis. The reasons for the formation of PPE include the interaction between inflammation and coagulation, as well as the occurrence of intrapleural fibrosis. Elevated level of D-dimer may represent microcirculation thrombosis or extracellular fibrin remodelling [39]. In this study, PPE patients have significant elevated D-dimer levels, indicating the disorder of coagulation and fibrinolysis. Multivariate logistic regression analysis revealed that serum albumin (OR = 0.876, 95%CI: 0.790–0.971, *P* = 0.012) and serum sodium (OR = 0.896, 95%CI: 0.828–0.969, *P* = 0.006) is also an independent risk factor for PPE in elderly patients with CAP, which is consistent with previous studies [20].

There were some limitations in this study. Firstly, being a retrospective observational study, the number of cases was relatively small, and some data were missing. Secondly, in some patients with pleural effusion, the B-ultrasound showed minimal effusion, making safe extraction of relevant laboratory indicators difficult, thereby leading to their exclusion from the analysis. Thirdly, due to the limited sample size, further stratified comparisons could not be conducted, and the accuracy and applicability of the results still needs to be confirmed through further in-depth research.

## Conclusion

As one of the most common complications of pneumonia, PPE poses an increasing problem in the elderly, leading to elevated morbidity and mortality. Early diagnosis and treatment are crucial for elderly patients with CAP. Proper selection of antibiotics, active management of underlying diseases, timely correction of hypoproteinaemia and electrolyte imbalances and prompt placement of adequate drainage can significantly reduce the length of hospital stay, lower the risk of complications and ultimately decrease mortality rates. This study aimed to analyse the risk factors of patients with PPE to provide an evaluation system for studying PPE patients in China, providing valuable guidance for further improvement of the clinical diagnosis and treatment through clinical validation, preventing further disease progression.

### Supplementary Information

**Additional file 1.**

## Data Availability

All data generated or analyzed during this study are included in this published article [and its supplementary information files].

## Abbreviations

CAP: Community-acquired pneumonia
PPE: Parapneumonic pleural effusion
NPPE: Non parapneumonic pleural effusion
CRP: C-reactive protein
PCT: Procalcitonin
IOR: Interquartile range
Year: Yr
CI: Confidence interval
